# The practice of venous blood collection among laboratory and non-laboratory professionals working in Ethiopian Government Hospitals: a comparative study

**DOI:** 10.1186/1472-6963-14-88

**Published:** 2014-02-25

**Authors:** Mulugeta Melkie, Abel Girma, Tsegaye Tsalla

**Affiliations:** 1 Department of Medical Laboratory Science, Arbaminch University, Arba Minch, Ethiopia

**Keywords:** Venous blood collection, Pre-analytical error, Ethiopia

## Abstract

**Background:**

Pre-analytical phase of overall laboratory testing system continues to be the major source of errors that affect patient safety and health care system. One of the activities in this phase is venous blood collection (VBC), the most common type of specimen drawn or sent to clinical laboratories for further analysis; and the source for a potentially numerous types of errors. In this study, we focused on determining and comparing desirability/undesirability of activities during VBC in Ethiopian hospitals among different groups of professionals.

**Methods:**

We conducted a cross-sectional comparative study in three government hospitals in South Ethiopia from February 2012 to September 2012. Randomly selected professionals who participate in VBC in outpatient and inpatient departments were requested to fill in structured and pretested questionnaire regarding their practice of VBC and their replies were categorized as ‘desirable’ and ‘undesirable’ according to Clinical and Laboratory Standards Institute (CLSI) standard. Then, data was analyzed using Medcalc® version 12.1.4 software. *P* value of less than 0.05 was considered as statistically significant.

**Results:**

In our study, 120 professionals were included among which 15.8% (n = 19) were laboratory professionals while the remaining 84.2% (n = 101) were non-laboratory professionals. Conscious patient identification in pre-collection phase of VBC and position of patients’ hands in actual collection phase of VBC involved the highest proportion of undesirability among both groups of professionals. However, in the post collection phase, specimen transferring from syringes to test tubes (15.8%) and mixing specimen with additives (63.4%) involved highest proportions of undesirability among laboratory and non-laboratory professionals respectively. Laboratory professionals reported better desirable practice in patient identification frequency, labeling and checking expiry dates of test tubes, specimen transfer and transport practices.

**Conclusion:**

In conclusion, preparatory activities of VBC involved the highest proportions of undesirable practices among both groups of professionals. However, relatively better proportions of desirability were seen among laboratory professionals than non-laboratory professionals in some pre- and post-collection phase activities. The difference might be seen as a result of better qualification, education and training experience on VBC among laboratory professionals.

## Background

Improving the quality of service provided by clinical laboratories is critical as the laboratory results affect up to 60-70% of clinical decisions [1]. Tremendous achievements have been accomplished regarding quality of the analytical phase as a result of entry of automations, advanced analytical techniques and sophisticated information technology, improvement in training and qualification of testing personnel, and establishment of effective external quality assessment schemes [1,2].

However, the improvement in the quality of pre-analytical phase has been sluggish and remains to be source of up to 80% of total laboratory errors [3,4]. This is due to the complex, time- and labor-intensive nature of the activities in this phase. The pre-analytical phase includes many error prone activities that could not be solved with the available technological advancements [5,6]. The errors originating from this phase have been reported to lead to further inappropriate investigations and unjustifiable increase in costs and, inappropriate care or inappropriate modifications to therapy [7]. The increase in turnaround time is also found to partly be resulted from unnecessary laboratory test repetitions that require patients to return to the specimen collection site for replacement [8,9].

In this study, we assessed and compared the proportions of desirability in activities of venous blood collection (VBC) among laboratory and non laboratory professionals carrying out VBC in outpatient and inpatient departments respectively. As it is described in literature, venous blood specimen (VBS) is the most common type of specimen drawn or sent to clinical laboratoies for further analysis; and is the source for a potentially numerous types of errors [3,10]. Moreover, differences have been noted on desirability of VBC activities when performed by different groups of professionals. In fact, many findings have been disclosed concerning the magnitude and types of pre-analytical errors committed during VBC. However, most of them were obtained from studies conducted in developed countries that are supposed to possess accredited laboratories and qualified personnel. In addition, the studies were focused on determining the magnitude of errors not on identifying activities causing the errors. Hence, the figures and the sources of pre-analytical errors might not signify the situation in developing countries including Ethiopia.

## Method

We conducted a cross-sectional study in three government hospitals (Arbaminch General Hospital, Chencha District Hospital and Sawula District Hospital) of Gamo Gofa zone, South Ethiopia from February 2012 to September 2012.

In the hospitals, VBC was performed in two ways; the needle and syringe method and the vaccutainer method using BD® needles and evacuated tubes. However, most of the professionals often tend to draw the blood using syringe and transfer it to evacuated tubes.

Questionnaire was prepared from the Clinical and Laboratory Standards Institute (CLSI) standard on procedures for the collection of diagnostic blood specimens by venipuncture (H3-A6, 2007) [11]. Laboratory professionals who perform VBC in laboratories (outpatient departments) and non-laboratory professionals who take part in VBC in wards (inpatient departments) were requested to fill in structured and pre-tested questionnaire regarding their practice of VBC. We determined desirability/undesirability of the activities in VBC by setting operational definitions from the same source we used to prepare the questionnaire.

The proposal of this study was ethically approved by the Institutional Ethical Review Committee (IRC) of Arbaminch University. Moreover, the hospitals were requested providing with the letter of ethical approval, for permission to use that health facility to conduct this research. Written informed consents from each study participants were also obtained after clear explanation about the objective and purpose of the study.

The data was entered and cleaned in a Microsoft Excel sheet and exported to Medcalc® version 12.1.4 software for further analyses. The data was summarized and presented in tables. Chi-square test and Fisher’s exact test were used to determine associations between profession and the desirability/undesirability of activities in VBC. *P* value of less than 0.05 was considered as statistically significant.

## Results

### Socio-demographic and background information about study participants

A total of 120 professionals were included in this study from the 3 government hospitals of Gamo Gofa zone, southern Ethiopia. The median age of study participants was 26 years (IQR = 24–30 years). Female study participants accounted for 45% (n = 54). 15.8% (n = 19) of the study participants were laboratory professionals who participate in VBC in laboratories (outpatient departments) while the remaining 84.2% (n = 101) were non-laboratory professionals who take part in VBC in wards (inpatient departments). With regard to their qualification, majority (89.2%, n = 107) were diploma holders and the 10.8% (n = 13) were first degree holders. The median years of experience among study participants was 4 years (IQR = 2–7 years).

Many of the study participants (64.2%, n = 77) responded that they have got an education about VBC during their stay in Colleges/Universities. Only 9.2% (n = 11) of study participants reported that they have got an on-job training on VBC. Table 1 summarizes the difference in the distribution of laboratory versus non-laboratory professionals with regard to the studied socio-demographic and background characteristics.

**Table 1:** Socio-demographic and background characteristics of study participants (n = 120); and comparison of the characteristics among laboratory (n = 19) and non-laboratory professionals (n = 101)

	***Overall frequency (%)***	***Profession***		
		***Laboratory professionals frequency (%)***	***Non-laboratory professionals frequency (%)***	***p*-value**
***Working site***				
Arbaminch general hospital	60 (50.0%)	10 (52.6%)	50 (49.5%)	0.967^§^
Chencha district hospital	34 (28.3%)	5 (26.3%)	29 (28.7%)	
Sawula district hospital	26 (21.7%)	4 (21.1%)	22 (21.8%)	
***Gender***				
Male	66 (55.0%)	15 (78.9%)	51 (50.5%)	0.025^*^
Female	54 (45.0%)	4 (21.1%)	50 (49.5%)	
***Age group***				
≤ 26 years	57 (47.5%)	11 (57.9%)	52 (51.5%)	0.793^§^
> 26 years	63 (52.5%)	8 (42.1%)	49 (48.5%)	
***Qualification***				
Diploma	107 (89.2%)	14 (73.7%)	93 (92.1%)	**0.050**^**§**^
First degree	13 (10.8%)	5 (26.3%)	8 (7.9%)	
***Experience***				
≤ 4 years	61 (50.8%)	13 (68.4%)	48 (47.5%)	0.155^§^
> 4 years	59 (49.2%)	6 (31.6%)	53 (52.5%)	
***Ever got education on *** **VBC**				
*Yes*	77 (64.2%)	19 (100%)	43 (42.6%)	<0.001^*^
*No*	43 (35.8%)	0 (0%)	58 (57.4%)	
***Ever got on-job training on *** **VBC**				
*Yes*	11 (9.2%)	5 (26.3%)	6 (5.9%)	**0.017**^**§**^
*No*	109 (90.8%)	14 (73.7%)	95 (94.1%)	

^§^Chi-square test, ^*^Fisher’s exact test, VBC = Venous Blood Collection.

For better understanding, we present the result of this study by categorizing activities of VBC as pre-collection, actual collection and post collection phases although the VBC by itself is one of the pre-analytical activities in the overall laboratory testing system. Hence, the pre-collection phase of VBC included practices related to patient identification, patient preparation and material preparations for the procedures. In the actual collection phase, we considered activities related to actual specimen collection while the post collection phase is consisted of activities such as specimen handling and transportation.

### The pre-collection phase

Overall, we found that 40% (n = 48) of the study participants did not have the desirable practice of identifying a patient as a routine task before the actual VBC. Only one-fifth of the study participants (20.8%, n = 25) have the desirable practice of identifying a conscious patient by asking to state his/her name and cross check it with the name mentioned on the request paper. With regard to unconscious patient identification, 59.7% (n = 71) responded desirable way of identifying such patient by asking the families of that patient to state patient’s name and cross check it with the name mentioned on the request paper. Information concerning diet restrictions prior VBC was only collected by 59.2% (n = 71) of participants. Only 40.8% (n = 49) of participants allowed patients to take the desirable duration of rest for 10 to 15 minutes before the beginning of VBC. Majority of the respondents (81.7%, n = 98) reported that they label test tubes although the information on the label was insufficient: 28.6%, 13.3%, 29.6% and 28.6% of participants label test tubes with patient’s first and last name, date and time of collection, patient’s hospital identification number, and all the afore mentioned information excluding the initial of the specimen collector respectively. Among all the study participants, 70% (n = 84) of them reported that they routinely check expiry dates of test tubes prior to collection.

As summarized in Table 2, the top three pre-collection phase activities that involved high proportions of undesirable practice among laboratory professionals were conscious patient identification (89.5%), diet restriction assessment (63.2%) and allowing patients to have seated rest (57.9%) respectively. On the other hand the top three pre-collection phase activities that involved high proportions of undesirable practice among non-laboratory professionals were conscious patient identification (77.2%), allowing patients to have rest in sitting position (59.4%) and patient identification frequency (47.5%).

**Table 2:** Comparison of undesirability of selected activities in the three phases of VBC among laboratory (n = 19) and non-laboratory professionals (n = 101)

	***Profession***		**Total**	***p*-value**
	***Laboratory professionals frequency (%)***	***Non-laboratory professional frequency (%)***		
***1. Pre-collection phase***				
***Patient identification frequency***				
Undesirable	0 (0%)	48 (47.5%)	48 (40%)	<0.001^*^
***Conscious patient identification***				
Undesirable	17 (89.5%)	78 (77.2%)	95 (79.2%)	0.357^*^
***Unconscious patient identification***				
Undesirable	8 (42.1%)	41 (40.6%)	49 (40.8%)	0.895^§^
***Diet restriction assessment***				
Undesirable	12 (63.2%)	37 (36.6%)	49 (40.8%)	**0.057**^**§**^
***Allowing patients to rest in sitting***				
Undesirable	11 (57.9%)	60 (59.4%)	71 (59.2%)	0.895^§^
***Labeling test tubes***				
Undesirable	0 (0%)	22 (21.8%)	22 (18.3%)	0.022^*^
***Checking expiry dates of test tubes***				
Undesirable	2 (10.5%)	34 (33.7%)	36 (30%)	0.056^*^
***2. Actual collection phase***				
***Vein selection procedure***				
Undesirable	6 (31.6%)	50 (49.5%)	56 (46.7%)	0.236^§^
***Wearing gloves***				
Undesirable	4 (21.1%)	12 (11.9%)	16 (13.3%)	0.281^*^
***Puncture site cleansing***				
Undesirable	6 (31.6%)	31 (30.7%)	37 (30.8%)	0.846^§^
***Wetness of the site during needle insertion***				
Undesirable	2 (10.5%)	29 (28.7%)	31 (25.8%)	0.151^*^
***Way of pulling syringe plunger out of veins***				
Undesirable	0 (0%)	4 (4.0%)	4 (3.3%)	1.000^*^
***Patients’ hand position during sampling***				
Undesirable	16 (84.2%)	65 (64.4%)	81 (67.5%)	0.113^*^
***Timing of tourniquet removal***				
Undesirable	12 (63.2%)	37 (36.6%)	49 (40.8%)	**0.057**^**§**^
***3. Post-collection phase***				
***Specimen transferring practice***				
Undesirable	3 (15.8%)	64 (63.4%)	67 (55.8%)	<0.001^*^
***Mixing specimen with additives***				
Undesirable	1 (5.3%)	64 (63.4%)	65 (54.2%)	<0.001^*^
***Specimen storage***				
Undesirable	1 (5.3%)	26 (25.7%)	27 (22.5%)	0.070^*^
***Specimen transport***				
Undesirable	2 (10.5%)	45 (44.6%)	47 (39.2%)	0.005^*^

^§^Chi-square test, ^*^Fisher’s exact test, VBC = Venous Blood Collection.

### The actual collection phase

In both categories 77.5% (n = 93) of professionals reported that they always apply tourniquets on patients regardless of visibility of veins while the remaining 22.5% (n = 27) of them apply tourniquets occasionally. Almost half of the study participant (46.7%, n = 56) reported undesirable way of selecting veins by simply inspecting visually without checking by palpation. Wearing gloves on routine basis during VBC was only reported by 86.7% of study participants (n = 104). With regard to cleansing the site of VBC, 69.2% (n = 83) of the respondents reported that they always clean the area by applying an antiseptic in a circular motion from center to periphery of the site. After the cleansing procedure, a quarter (25.8%, n = 31) of the study participants reported undesirable practice of inserting the needle in to the vein without waiting until the antiseptic dries. Small proportion of the study participants (3.3%, n = 4) reported undesirable practice of syringe plunger pulling by forceful and speedy way. 67.5% of study participants (n = 81) reported undesirable practice regarding hand condition of patients (i.e. clenched, pumping) during VBC. Concerning tourniquet removal, 59.2% (n = 71) of the participants reported the desirable practice of removing tourniquets after needle insertion in to the vein of the patient and assurance of blood reach at the hub of the needle.

Activities in the actual collection phase that involved highest proportions of undesirable practice among laboratory professionals include patients’ hand condition during sampling (84.2%) and timing of tourniquet removal (63.2%) while patients’ hand condition during sampling (64.4%) and vein selection procedure (49.5%) involved highest proportions of undesirable practice among non-laboratory professionals (Table 2).

### The post collection phase

Overall, desirable practice of transferring blood from the barrel of the syringe to test tubes by removing the needle first and gently ejecting the blood against the wall of the test tube was reported by 44.2% (n = 53) of study participants. After pouring the blood into test tubes with additives, 46.2% (n = 55) of study participants responded that they mix the blood with the additive immediately using the desirable way of mixing by several gentle inversions. Concerning storage of the collected VBS, 77.5% (n = 97) of study participants responded that they put the test tube vertically in a rack while the remaining 22.5% (n = 23) of them reported undesirable practices of storing VBS like simply laying it on tables or putting it in pockets of their gowns. 60.8% (n = 73) of study participants reported the desirable practice in transporting collected VBS to laboratory by using racks avoiding direct sun light exposure while the rest 39.2% (n = 47) of them failed to do so.

Specimen transferring from syringes to test tubes (15.8%, n = 3) and transporting (10.5%, n = 2) activities were the post collection phase activities that involved the highest proportions of undesirable practice among laboratory professionals. On the other hand, mixing specimens with additives (63.4%, n = 64) and specimen transferring from syringes to test tubes (63.4%, n = 64) were activities that involved the highest proportions of undesirable practice among laboratory professionals in this phase (Table 2).

## Discussion

In our study, we categorized activities in VBC process into pre-collection, actual collection and post collection phases although VBC by itself can be considered as part of pre-analytical phase when the total testing system is taken as a reference [4]. Surprisingly, as in the case of total testing system where in pre-analytical phase activities were reproducibly found to be the major sources of errors [4], we found that the pre-collection phase activities of VBC involved the highest proportions of undesirable undertakings by study participants irrespective of profession followed by post-collection and actual collection phase activities. This can give an impression that emphasis is not given to preparatory activities and activities after the undertaking of actual work in laboratories.

### Pre-collection phase

We considered the patient identification practice by study participants as the first key element in the pre-collection phase since it is obligatory if a VBS is intended to be collected from the right person who is designated on the request paper. In this regard, the laboratory professionals we studied were better than the non-laboratory professional (*p < 0.001*) in terms of confirming patients’ identities on routine basis. The malpractice reported by the non-laboratory professionals in inpatients might be resulted from ‘I already know the patient’ sense as indicated by Wallin et al. and lack of knowledge and attitude [3,12].

However, the approach followed by both groups of professionals to confirm identity of patients (both conscious and unconscious) was prone to misidentifications and subsequent pre-analytical errors. These could have serious consequences in the clinical decision-making process and may affect patient safety. It has been reported that patient identification errors were responsible for about 8.8% of all laboratory errors [9] and for more than 25% of all pre-analytical errors; and the mistakes were associated with repeated specimen collection, or repeated laboratory analyses thus resulting in an unjustified increase in costs [8,13]. Occasionally, such errors also could result death of the patient [14,15].

Allowing patients to have rest in sitting position prior VBC was another activity that we determined high (*p = 0.895*) and approximate proportions of undesirability in both groups of professionals. As a standard practice, both lying down and ambulatory patients need to have sufficient sitting rest before VBC. Insufficient sitting rest of ambulatory patients before collection allows escape of fluid into the interstitial space. Thus, components that cannot pass the vessel wall, mainly proteins, will increase in concentration. On the contrary, insufficient sitting rest of patients that are in supine position allows accumulation of extravascular fluid in the blood vessels which results in hemo-dilution and subsequent decrease in certain laboratory parameters [16].

Diet is one of the variables to be monitored prior VBC since quality/suitability of the specimen and some of the laboratory tests could be affected otherwise. To guide this, CLSI and International Standards Organization (ISO) recommended institutionalizing and strict adherence to pre-analytical manual, a document that provides directions on the instructions given to patients prior to specimen collection [11,17]. In this study, non-laboratory professionals reported better desirable practice with regard to diet restriction assessment than laboratory professionals though it was not statistically significant (*p = 0.057*). This might emanate from the fact that ensuring proper patient preparation including diet restriction is not responsibility of only professionals collecting the specimen but also of patients and clinicians [3,18]. Hence, the non-laboratory professionals we studied were presumed to work closely with clinicians and seem to have better control of their patients than laboratory professionals. These might enabled them to grasp prior preparation instructions given by clinicians and subsequently help patients to comply with the instructions [19].

Although it was not statistically significant, non-laboratory professionals reported better desirable practice of routinely checking expiry dates of test tubes prior VBC than non-laboratory professionals (*p = 0.056*).

In addition to routine inspection of expiry dates of test tubes, the laboratory professionals reported better desirable practice regarding test tubes labeling during VBC than non-laboratory professionals (*p = 0.022*). However, the labels they reported to made on test tubes contained insufficient information about the specimen that might lead to identification errors. It has been reported that 50% of all identification errors in laboratories occurred as a result of malpractices relater to labeling [20]. Hence, proper test tube labeling is utmost importance for patient safety and it should always be done during VBC [21,22]. The labels should be firmly attached on to test tubes bearing at least the following information: the patient’s first and last names, an identification number, the date and time of collection and the identification of the person collecting specimen [11]. The labels are better written using computers to avoid legibility problems when made by hand writings.

### Actual collection phase

In this study, we did not get statistically significant associations between profession and activities in the actual collection phase. Nevertheless, proportions of undesirability of actual collection phase activities among laboratory professionals were second highest followed by post collection activities; while undesirability of post collection phase activities were second highest followed by actual collection phase activities among non-laboratory professionals.

We determined that failure of both groups of professionals to instruct and/or provision of misguiding information to patients regarding hand positions during VBC was the leading malpractice in the actual collection phase followed by improper vein selection procedure, inappropriate timing of tourniquet removal, improper puncture site cleansing, failure to wait until puncture site dries after application of an antiseptic, failure to wear gloves and inappropriate way of pulling syringe plunger out of veins.

Some of these malpractices have been reported to affect the quality of venous blood specimen in terms of causing hemolysis. Hemolysis is a very common error that accounts for more than 50% of all pre-analytical errors [3,8,18,19]. It can affect various tests due to the lysis of erythrocytes and subsequent release of erythrocytic contents and also, the reddish color of the serum/plasma may interfere with various assays [23]. If the antiseptic applied to cleanse the puncture site is not allowed to completely dry, it might be contaminated with the specimen and lead to hemolysis [24]. In addition, undesirable practices reported by both groups of professionals regarding improper vein selection procedure (by visual inspection only) that might lead to excessive probing to find a vein and inappropriate way of pulling syringe plunger out of veins in a speedy and forceful way, could cause hemolysis [23,24].

Failure to remove tourniquets after needle insertion in to the vein of the patient and assurance of blood reach at the hub of the needle could also cause hemolysis as a result of prolonged application [25,26]. Besides hemolysis, it could induce venous stasis that promotes exit of water, diffusible ions and low molecular weight substances from the vessel thereby increasing the concentration of various blood analytes at the punctured site thus potentially influencing the laboratory results interpretation. Moreover, it brings the pH of the blood to be lower as a result of tissue hypoxia which in turn affects many laboratory parameters [27]. However, if tourniquet is not applied for more than 60 seconds, the impact on values of almost all laboratory parameters can be neglected and is even below the tolerable amount random errors [28].

We were surprised to learn that wearing gloves during VBC was not practiced on routine basis by both groups of professionals (*p = 0.281*). Together with the malpractice in the cleansing procedure followed by professionals, this would hamper patient safety during VBC.

### Post collection phase

We noted statistically significant differences in the proportions of undesirability on activities identified in this phase of VBC among studied groups of professionals. Laboratory professionals reported better proportions of desirable practices than non-laboratory professionals with regard to specimen transferring from the barrel of the syringe in to test tubes (*p < 0.001*), specimen transport (*p = 0.005*) and specimen mixing with additives (*p < 0.001*). Although it was not statistically significant, we also found similar finding concerning specimen storage practice (*p = 0.070*) after VBC. This was in agreement with another study in terms of showing higher proportion of undesirable practice of VBS storage among non-laboratory professionals than laboratory professionals [12]. Undesirable practices of storing VBS in a way of simply laying them on tables or putting them in pockets of ones gowns might disturb clot formation if the specimen is intended for further processing to produce serum. The inadequacy in clot formation in turn may result in small volume of serum that might force laboratory personnel to perform rimming/dislodging tubes with wooden applicator stick, an undesirable procedure that make specimens prone to hemolysis [29].

When VBS is collected in test tubes containing additives, anticoagulants in our case, it is recommended to adequately mix the specimen with the additive immediately after VBC. Inadequate and/or late inversion of test tubes can cause clotting of the blood which has been reported to constitute an important part of pre-analytical errors in VBC [8,19]. Nevertheless, care should be taken not to vigorously shake the test tubes as it may result in hemolysis [23]. Yet the non-laboratory professionals in our study failed to report an immediate and/or gentle inversion of test tubes to mix VBS with the anticoagulant. They were also unable to meet requirements that we considered, putting test tubes in racks and light exposure, to assure specimen stability during transportation from collection center to laboratory.

Statistically significantly high proportion of undesirable practice regarding specimen transfer in to test tubes after VBC was noted among non-laboratory than laboratory professionals (*p < 0.001*). This improper technique which is characterized by pouring blood into tubes through the needle and with great pressure forming froth would cause errors like hemolysis [23,24].

Generally, laboratory professionals reported better desirable practice in some pre- and post-collection phase activities of VBC than non-laboratory professionals. However, we were not bold enough to say that these variations came as a result of socio-demographic and background characteristics differences among the groups like gender, qualification, accessing education and training on VBC. That is because, if these differences were responsible for making pre- and post-collection phase practice of laboratory professionals more desirable than practice of non-laboratory professionals, we could have been able to get similar effects on actual collection phase activities. So, besides training and educating health professionals on VBC, encouraging them to develop and adhere to standard operating procedure (SOP) that accommodates all aspects of VBC would minimize the proportion of undesirable practices among health professionals irrespective of profession.

As a limitation to this study, we measured proportions of desirability of VBC activities based on self-reports by study participants. We were unable to carryout observational studies which might give us opportunities to comprehend actual practices and give us more flexibility in picking up error prone activities during the process of VBC by our study participants.

## Conclusion

In conclusion, pre-collection phase activities involved the highest proportions of undesirable practices among both groups of professionals. However, relatively better proportions of desirability were seen among laboratory professionals than non-laboratory professionals in some pre- and post-collection phase activities. The difference might be seen as a result of better qualification, education and training experience on VBC among laboratory professionals.

## Competing interests

The authors declared that they have no any relevant competing interest to disclose in this research.

## Authors’ contributions

MM has conceived and designed the study. MM, AG, and TT have participated in acquisition of data. MM, AG, and TT have participated in preparing and critically reviewing the draft manuscript. All authors have read and approved the final manuscript.

## Pre-publication history

The pre-publication history for this paper can be accessed here:

http://www.biomedcentral.com/1472-6963/14/88/prepub
